# Characterization of Somatic Mutations That Affect Neoantigens in Non-Small Cell Lung Cancer

**DOI:** 10.3389/fimmu.2021.749461

**Published:** 2022-03-09

**Authors:** Hongge Liang, Yan Xu, Minjiang Chen, Jing Zhao, Wei Zhong, Xiaoyan Liu, Xiaoxing Gao, Shanqing Li, Ji Li, Chao Guo, He Jia, Mengzhao Wang

**Affiliations:** ^1^ Department of Respiratory and Critical Care Medicine, Peking Union Medical College Hospital, Chinese Academy of Medical Sciences and Peking Union Medical College, Beijing, China; ^2^ Department of Respiratory and Critical Care Medicine, Peking University People’s Hospital, Beijing, China; ^3^ Department of Thoracic Surgery, Peking Union Medical College Hospital, Chinese Academy of Medical Sciences and Peking Union Medical College, Beijing, China; ^4^ Department of Pathology, Peking Union Medical College Hospital, Chinese Academy of Medical Sciences and Peking Union Medical College, Beijing, China; ^5^ Department of Biological Information, Beijing Neoantigen Biotechnology Co., Ltd., Beijing, China

**Keywords:** non-small cell lung cancer, whole exome sequencing, neoantigens, tumor neoantigen burden, genetic mutation characteristics

## Abstract

**Purpose:**

Immune checkpoint inhibitors (ICIs) have recently emerged as an important option for treating patients with advanced non-small cell lung cancer (NSCLC). Neoantigens are important biomarkers and potential immunotherapy targets that play important roles in the prognosis and treatment of patients with NSCLC. This study aimed to evaluate and characterize the relationships between somatic mutations and potential neoantigens in specimens from patients who underwent surgical treatment for NSCLC.

**Patients and Methods:**

This prospective study evaluated specimens from patients with NSCLC who underwent surgical treatment at the Peking Union Medical College, China, from June 2019 to September 2019. Whole-exome sequencing was performed for tumor tissues and corresponding normal tissues. Candidate neoantigens were predicted using generative software, and the relationships between various mutation characteristics and number of neoantigens were evaluated.

**Results:**

Neoantigen-related gene mutations were less frequent than mutations affecting the whole genome. Genes with high neoantigen burden had more types and higher frequencies of mutations. The number of candidate neoantigens was positively correlated with missense mutations, code shift insertions/deletions, split-site variations, and nonsense mutations. However, in the multiple linear regression analysis, only missense mutations were positively correlated with the number of neoantigens. The number of neoantigens was also positively correlated with base transversions (A>C/C>A, T>G/G>T, and C>G/G>C) and negatively correlated with base transitions (A>G/G>A and C>T/T>C).

**Conclusion:**

The number of candidate neoantigens in NSCLC specimens was associated with mutation frequency, type of mutation, and type of base substitution.

## Introduction

Despite advances in treatment strategies during the last 20 years, lung cancer remains the leading cause of cancer-related deaths worldwide. Immune checkpoint inhibitors (ICIs) are antibody-derived molecules that have recently emerged as treatment options for many types of cancers. They target regulatory receptors such as cytotoxic T-lymphocyte associated protein 4, programmed cell death 1 (PD-1), and programmed cell death-ligand 1 (PD-L1). These treatments have provided significant clinical benefits and changed the treatment landscape for patients with advanced non-small cell lung cancer (NSCLC). Although ICIs used in first-line and second-line treatments provide survival advantages compared with chemotherapy, the objective response rate is only approximately 20% among unselected patients (1–11). Thus, it is important to effectively select patients who are expected to benefit from ICI treatment.

Currently, the only approved biomarker for predicting response to ICI treatment is PD-L1 expression. However, patients with low tumor expression of PD-L1 can still experience a treatment response, suggesting that PD-L1 is not entirely effective for selecting patients to receive immunotherapy (12). Other potential biomarkers for guiding ICI treatment include major histocompatibility complex (MHC) expression, lymphocyte count, tumor T-cell marker expression, tumor burden (TMB), and the neoantigen expression (13). Among the potential biomarkers, mutation-derived neoantigens have attracted considerable attention. These tumor cell-specific mutant peptides can be presented by MHC molecules (14, 15) and recognized by T cells. Thus, neoantigens can mediate the immune response to tumor cells (14, 15) and allow the host immune system to recognize and destroy them.

Recent advances in genomics and bioinformatics have laid the foundation for the effective selection of the strongest immunogenic neoantigens based on the tumor’s spectrum of somatic mutations. However, there are few reports regarding neoantigen-associated gene mutations in NSCLC. This information could be useful in identifying patients who might benefit from ICI treatment. Therefore, this study aimed to evaluate and characterize the relationship between somatic mutations and potential neoantigens in specimens from patients who underwent surgical treatment for NSCLC.

## Patients and Methods

### Patients

We prospectively collected patients with NSCLC who underwent surgical treatment at the thoracic surgery department of Peking Union Medical College Hospital between June 2019 and September 2019. The inclusion criteria were histopathologically confirmed NSCLC, complete clinical and pathological data, sufficient tumor and corresponding normal tissue for whole-exome sequencing, and provision of informed consent by the patient for the research use of their specimens for research purposes. Data such as sex, age, smoking history, histological type, TNM stage, and clinical stage were collected. The study protocol was approved by our institutional review board.

### Neoantigen Prediction

**Figure 1:**
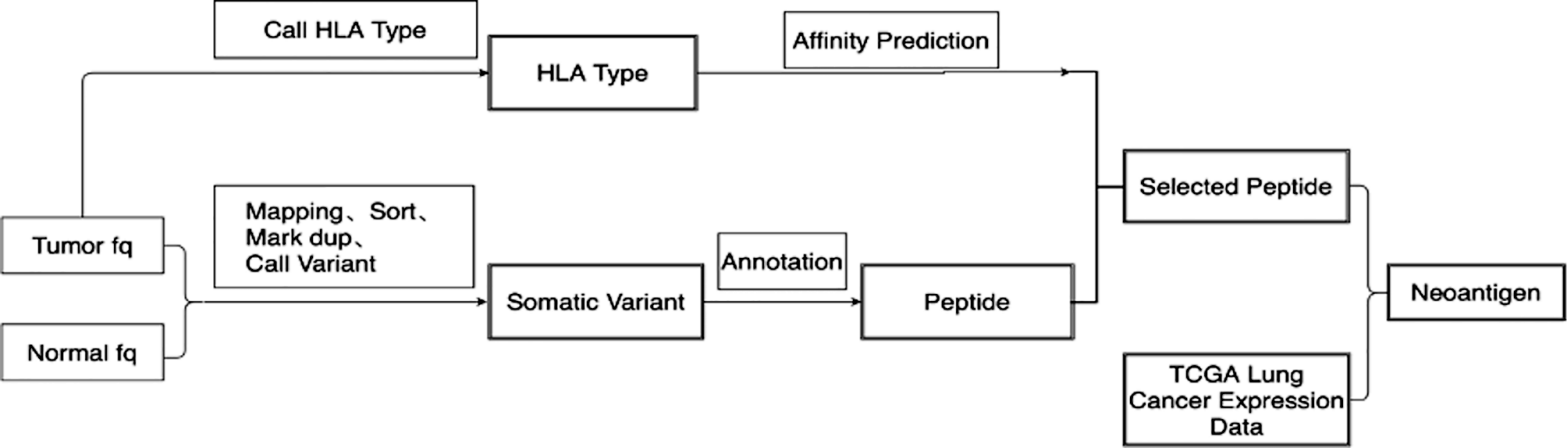
Flow chart of candidate neoantigen design. 1) Quality control and statistical analysis were performed on original tumor and normal FASTQ data. 2)Bwa was used to compare FASTQ data to human reference genome HG19. Samblaster was used to mark duplication on reads. 3)SamTools was used to convert the Sam file into BAM file and build index. 4)GATK Mutect2 was used to call and filter somatic mutation, and then obtain mutation information. 5)The original FASTQ information was used for HLA typing using Optitype. 6) Netmhcpan4 was used to predict peptide affinity according to HLA type. 7) The expression data of lung cancer patients in TCGA were downloaded, and the mean values of expression data of lung adenocarcinoma and lung squamous cell carcinoma patients were calculated respectively. 8) Neoantigen was scored according to the sequencing data, mutation frequency, expression of HLA, expression of transcript, expression of immune-related gene to obtain the final neoantigen results.

**Figure 2:**
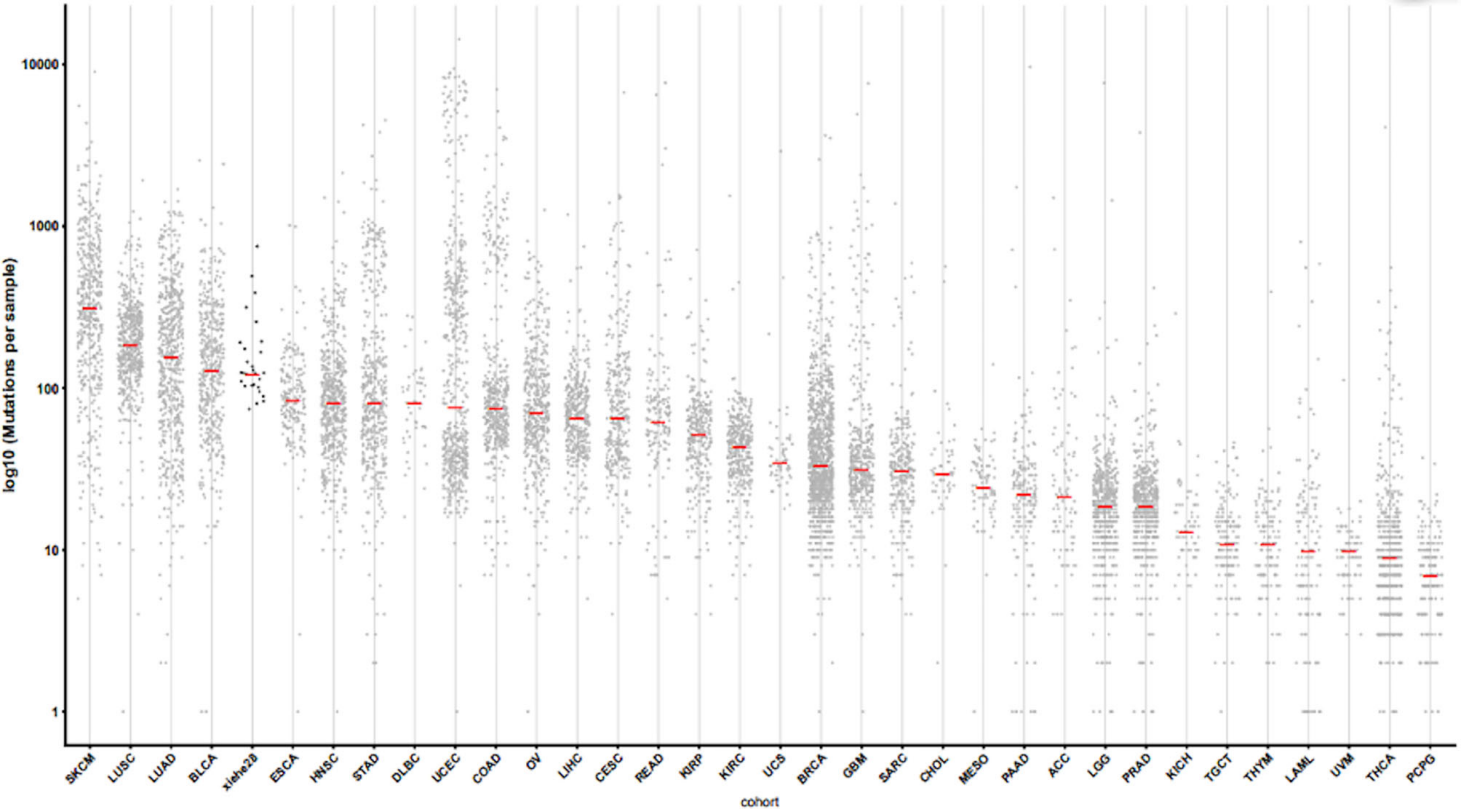
Gene disturbance map. The X-axis represents the sample type, and “xiehe28” is our 28 samples, and the other samples are tumors in TCGA database. The Y-axis represents log 10 (mutations per sample). The red line in the figure represents the mean value. Note, SKCM, skin cutaneous melanoma. LUSC, lung squamous cell carcinoma. LUAD, lung adenocarcinoma. BLCA, bladder cancer; ESCA, esophageal cancer; HNSC, head and neck squamous cell carcinoma; STAD, stomach adenocarcinoma; DLBC, Diffuse large B-cell lymphoma; UCEC, Uterine corpus endometrial carcinoma; COAD, colon adenocarcinoma; OV, Ovarian cancer; LIHC, liver hepatocellular carcinoma; CESC, cervical squamous cell carcinoma; READ, rectal adenocarcinoma; KIPP, Kidney renal papillary cell carcinoma; KIRC, kidney renal clear cell carcinoma; UCS, uterine carcinosarcoma; BRCA, breast cancer; GBM, glioblastoma; SARC, sarcoma; CHOL, cholangiocarcinoma; MESO, mesothelioma; PAAD, pancreatic adenocarcinoma; ACC, adrenocortical carcinoma; LGG, lower grade glioma; PRAD, Prostate adenocarcinoma; KICH, kidney chromophobe; TGCT, tenosynovial giant cell tumor; THYM, thymoma; LAML, acute myeloid leukemia; UVM, uveal melanoma; THCA, thyroid carcinoma; PCPG, pheochromocytoma and paraganglioma.

Whole-exome sequencing results were obtained for tumor tissues and corresponding normal tissues. Neopipe software was then used to predict candidate neoantigens by combining gene expression with the molecule’s predicted affinity for class I MHC (**Figure 1**). Data on RNA expression were not obtained because of financial constraints. However, gene expressions were referenced from the TCGA database for NSCLC (**Figure 2**). For lung adenocarcinoma and lung squamous cell carcinoma, the mean values for transcriptional and genetic quantification were collected from 574 lung adenocarcinoma cases and 548 lung squamous cell carcinoma cases. For all other pathological types, the mean values for transcriptional and genetic quantification were collected from all NSCLC cases.

To facilitate future research regarding *in vitro* synthesis and administration, neoantigens were designed as 25-mer peptides. Mutated 8–11-mer peptides that could bind to MHC were defined as neoepitopes. Given the difference in neoantigen epitopes and MHC affinities, the accuracy of predicting immune stimulation would be low if it was based only on the number of neoantigen epitopes. Therefore, the main results included the neoantigen-related gene mutation characteristics without considering neoepitopes.

### Statistical Analysis

All analyses were performed using SPSS software (version 21.0; IBM Corp., Armonk, NY, USA). Results are reported as median, number, frequency, and composition ratio, as appropriate. Nonparametric tests were used to analyze clinicopathological features associated with the number of neoantigens. Spearman’s test was used to analyze the correlation between neoantigens and gene mutation characteristics. Multiple linear regression analysis was conducted for the various neoantigen-related gene mutation types. Heat maps were created, and related cluster analyses were performed using Rstudio software. Using a bilateral test, results were considered statistically significant at P <0.05.

## Results

### Clinicopathological Features and Prediction of Candidate Neoantigens

Between June and September 2019, 34 patients underwent surgery for NSCLC at our center. We excluded 6 patients because of insufficient tissue samples or incomplete clinical data. Therefore, 28 patients were ultimately included in the study. The median age was 60.5 years (range: 38–76 years); 17 patients (60.7%) were men, and 15 patients (53.6%) had a history of smoking. The pathological types were adenocarcinoma (24 cases, 85.7%), squamous cell carcinoma (3 cases, 10.7%), and large-cell neuroendocrine carcinoma (1 case, 3.6%). The tumors were classified as stage I (19 patients, 67.9%), stage II (6 patients, 21.4%), and stage III (3 patients, 10.7%). Six patients (21.4%) had a family history of tumors (**Table 1**).

**Table 1:** Clinicopathological characteristics of 28 patients.

No.	Gender	Age	Smoking history (pack years)	Pathology	TNM stage	Clinical stage	Tumor size	Tumor history
**1**	Male	38	No	A	T1bN0M0	Ia2	14mm	No
**2**	Male	61	20	A	T1bN0M0	Ia2	23mm	No
**3**	Male	59	No	A	T1aN0M0	Ia1	10mm	No
**4**	Male	58	30	A	T1aN0M0	Ia1	8mm	No
**5**	Female	70	No	A	T1bN0M0	Ia2	20mm	No
**6**	Female	76	No	A	T1bN0M0	Ia2	20mm	Yes
**7**	Male	56	30	A	T1bN1M0	IIb	20mm	No
**8**	Male	70	10	A	T2aN0M0	Ib	40mm	No
**9**	Male	59	No	A	T1cN0M0	Ia3	25mm	No
**10**	Male	47	30	A	T2bN2M0	IIIa	20mm	No
**11**	Female	52	No	A	T1aN0M0	Ia1	10mm	No
**12**	Female	41	2	A	T3N2M0	IIIb	20mm	No
**13**	Female	60	No	A	T1cN0M0	Ia3	18mm	No
**14**	Female	68	No	A	T1bN0M0	Ia2	15mm	No
**15**	Male	73	No	A	T1cN0M0	Ia3	30mm	Yes
**16**	Female	60	No	A	T1bN0M0	Ia2	13mm	No
**17**	Female	54	3	A	T1cN0M0	Ia3	27mm	Yes
**18**	Female	71	No	A	T1bN0M0	Ia2	15mm	No
**19**	Female	58	No	A	T3N0M0	IIb	60mm	No
**20**	Female	63	No	A	T1bN0M0	Ia2	20mm	No
**21**	Male	49	30	S	T2aN1M0	IIb	32mm	No
**22**	Male	61	40	A	T1bN0M0	Ia2	15mm	Yes
**23**	Male	61	35	A	T2bN1M0	IIb	40mm	No
**24**	Male	56	30	A	T2bN2M0	IIIa	28mm	No
**25**	Male	63	40	A	T2aN0M0	Ib	36mm	Yes
**26**	Male	64	50	S	T1cN1M0	IIb	30mm	No
**27**	Male	64	3	S	T2bN1M0	IIb	60mm	Yes
**28**	Male	64	40	LCNEC	T2aN0M0	Ib	40mm	No

A, adenocarcinoma; S, squamous carcinoma; LCNEC, large-cell-neuroendocrine carcinoma.

**Figure 3:**
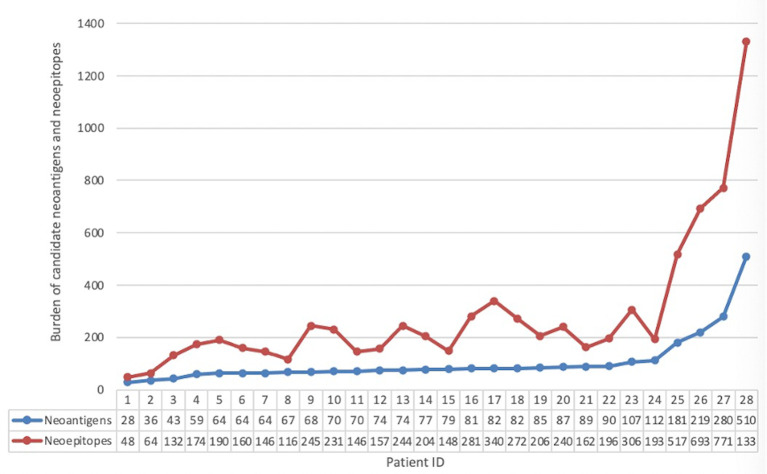
Neoepitope and neoantigen maps of the patients included in our study.

Whole-exome sequencing was performed for 28 NSCLC specimens, which identified 5,017 non-synonymous mutations, including 4,037 missense mutations, 419 frame-shift insertions/deletions, 313 in-frame insertions/deletions, 229 nonsense mutations, 10 non-stop mutations, and 9 splice site mutations. A total of 7,452 single-nucleotide variants, including A>T/T>A (n=539), A>C/C>A (n=966), A>G/G>A (n=2,006), T>C/C>T (n=1,990), T>G/G>T (n=1,025), and C>G/G>C (n=926), were identified. Using results from the 28 specimens, the Neopipe software predicted a total of 2,942 neoantigens (median: 78, range: 28–510) and 7,912 neoepitopes (median: 200, range: 48–1,300) (**Figure 3**). Spearman’s correlation analysis revealed a positive correlation between the tumor’s longest diameter and the number of predicted neoantigens (correlation coefficient=0.575, P=0.001). Additionally, the number of candidate neoantigens was higher among patients with a family tumor history (rank mean: 20.42 *vs*. 12.89, P=0.046) and patients with squamous cell carcinoma (rank mean: 26.47 *vs*. 13.28, P=0.019) (**Table S1**).

Nine of the twenty-eight patients had EGFR-sensitive mutations, including six cases with 21L858R, two cases with 19DEL, and one with 20INS. Nonparametric testing revealed that the number of candidate neoantigens was not correlated with EGFR mutations (P =0.087) (**Table S1**). One patient had EML4-ALK fusion (85 candidate neoantigens) and one patient had ROS1 fusion (36 candidate neoantigens). Three patients had KRAS mutations, including KRAS G12D mutation (87 neoantigens), KRAS G12D mutation combined with CDKN2A D108H mutation (28 neoantigens), and KRAS G12V mutation combined with TP53 K132E mutation and STK11 N181Y mutation (85 neoantigens).

### Gene Mutation Characteristics Associated With Candidate Neoantigens

**Figure 4:**
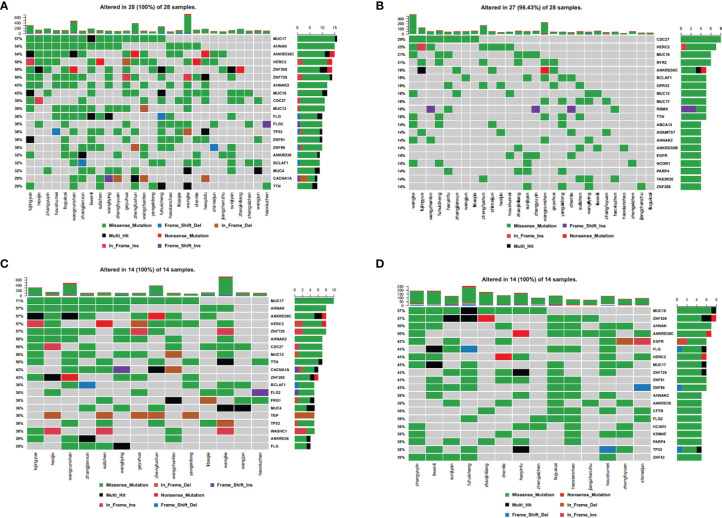
Spectral heat map of common gene mutations. **(A)** all mutations; **(B)** neoantigen-associated mutations. Spectrum heat map of common gene mutations in patients. **(C)** with high tumor neoantigen burden; **(D)** with low tumor neoantigen burden.

We analyzed whole genome mutations and neoantigen-related gene mutations and found revealed that the ten most commonly mutated genes were MUC17 (57%), AHNAK (54%), ANKRD36C (54%), HERC2 (50%), ZNF208 (50%), ZNF729 (50%), AHNAK2 (43%), MUC16 (43%), CDC27 (39%), and MUC12 (39%) (**Figure 4A**). The ten most commonly mutated neoantigen-related genes were CDC27 (29%), HERC2 (25%), MUC16 (21%), ANKRD36C (21%), BCLAF1 (18%), GPR32 (18%), MUC12 (18%), MUC17 (18%), PBMX (18%), and TTN (18%) (**Figure 4B**). Among the whole genome mutations, higher frequencies and more types of mutations were observed, including missense mutations, nonsense mutations, in-frame deletions, frame-shift deletions, in-frame insertions, frame-shift insertions, and mixed mutations. Conversely, the most common type of neoantigen-related gene mutations only involved missense mutations, nonsense mutations, in-frame insertions, frame-shift insertions, and mixed mutations.

### Comparing Mutation Characteristics According to Neoantigen Load

We defined tumor neoantigen burden (TNB) as the total number of neoantigens per million bases (Mbs) in a tumor sample. Using the median value (n=14), patients were assigned to a high TNB group (n>14) and a low TNB group (n ≤ 14), and the gene mutation characteristics were compared. In the high TNB group, the ten most commonly mutated genes were MUC17 (17%), AHNAK (57%), ANKRD36C (57%), HERC2 (57%), ZNF729 (57%), AHNAK2 (50%), CDC27 (50%), MUC12 (50%), TTN (50%), and CACNA1A (43%) (**Figure 4C**). In the low TNB group, the ten most commonly mutated genes were MUC16 (57%), ZNF208 (57%), AHNAK (50%), ANKRD36C (50%), EGFR (43%), FLG (43%), HERC2 (43%), MUC17 (43%), ZNF729 (43%), and ZNF91 (43%) (**Figure 4D**). Patients with high TNB had a higher frequency of gene mutations and more mutation types, including missense mutations, nonsense mutations, in-frame deletions, frame-shift deletions, in-frame insertions, frame-shift insertions, and mixed mutations. Furthermore, patients with high TNB had significantly more frequent mixed mutations, insertion mutations, and deletion mutations than patients with low TNB. Patients with low TNB had missense mutations, nonsense mutations, in-frame deletions, frame-shift deletions, in-frame insertions, and mixed mutations.

Therefore, neoantigen-related mutations had fewer mutation types than whole genome mutations, indicating that tumor neoantigen burden (TNB) is related to both the number and type of gene mutations. Patients with a high TNB also had more types of gene mutations than those with low TNB, which indicates that some mutation types may create a higher neoantigen load.

### Effects of Different Gene Mutation Types on Candidate Neoantigens

Spearman’s correlation analysis was performed to access the features of neoantigen-related gene mutations (**Table 2**). The number of neoantigens was positively correlated with the number of non-synonymous mutations (correlation coefficient=0.641, P <0.001). All non-synonymous mutation types were subsequently annotated and analyzed, and the number of neoantigens was positively correlated with missense mutations (correlation coefficient= 0.603, P <0.001), frame-shift insertions/deletions (correlation coefficient=0.755, P <0.001), nonsense mutations (correlation coefficient=0.501, P =0.007), and splice site mutations (correlation coefficient=0.546, P =0.003) (**Table 2**). These four mutation types were included in a multiple linear regression analysis, and the neoantigen number was only positively correlated with missense mutations (beta=0.674, P <0.001) (**Table 3**). This may be related to the high frequency of these mutations. Moreover, the lack of statistical significance between the number of candidate neoantigens and other mutation types might be related to their rarity, although they may produce a greater neoantigen load. The correlation between base substitution and number of neoantigens was also analyzed using Spearman’s correlation analysis. The number of neoantigens was positivity correlated with the following base transversions: A>C/C>A (correlation coefficient=0.641, P <0.001), T>G/G>T (correlation coefficient=0.388, P=0.041), and G>C/C>G (correlation coefficient=0.418, P=0.027). The number of neoantigens was negatively correlated with base transitions: A>G/G>A (correlation coefficient=-0.690, P<0.001) and T>C/C>T (correlation coefficient=-0.535, P=0.003) (**Table 2**).

**Table 2:** Spearman correlation analysis of candidate neoantigens.

Neoantigens	N	Correlation coefficient	P-value
nonsynonymous mutation	28	0664	<0.001
Frame shift indel	28	0.755	<0.001
In frame indel	28	0.071	0.718
Missense mutation	28	0.603	0.001
Nonsense mutation	28	0.501	0.007
Nonstop mutation	28	0.211	0.282
Splice site	28	0.546	0.003
A>T/T>A mutation frequency	28	0.279	0.151
A>C/C>A mutation frequency	28	0.641	<0.001
A>G/G>A mutation frequency	28	-0.690	<0.001
T>C/C>T mutation frequency	28	-0.535	0.003
T>G/G>T mutation frequency	28	0.388	0.041
C>G/G>C mutation frequency	28	0.418	0.027

**Table 3:** Multiple linear regression of candidate neoantigens.

Variants	Unstandardized coefficients		Unstandardized coefficients Beta	t-value	P-value	95.0% confidence interval of B	
	B	Standard error				Lower limit	Higher limit
(Constant)	-2.811	10.84		-0.259	0.798	-25.235	19.614
Frameshift indel	1.612	1.139	0.154	1.415	0.17	-0.744	3.968
Missense mutation	0.5	0.115	0.674	4.342	0	0.262	0.739
Nonsense mutation	0.782	1.418	0.08	0.552	0.587	-2.152	3.716
Splice site	16.209	12.101	0.104	1.339	0.194	-8.825	41.243

Therefore, the number of candidate neoantigens was related to both the number and type of mutations present in tumors and was also positively correlated with base transversions and negatively correlated with base transitions.

### Gene Mutations Associated With Multiple Candidate Neoantigens

**Figure 5:**
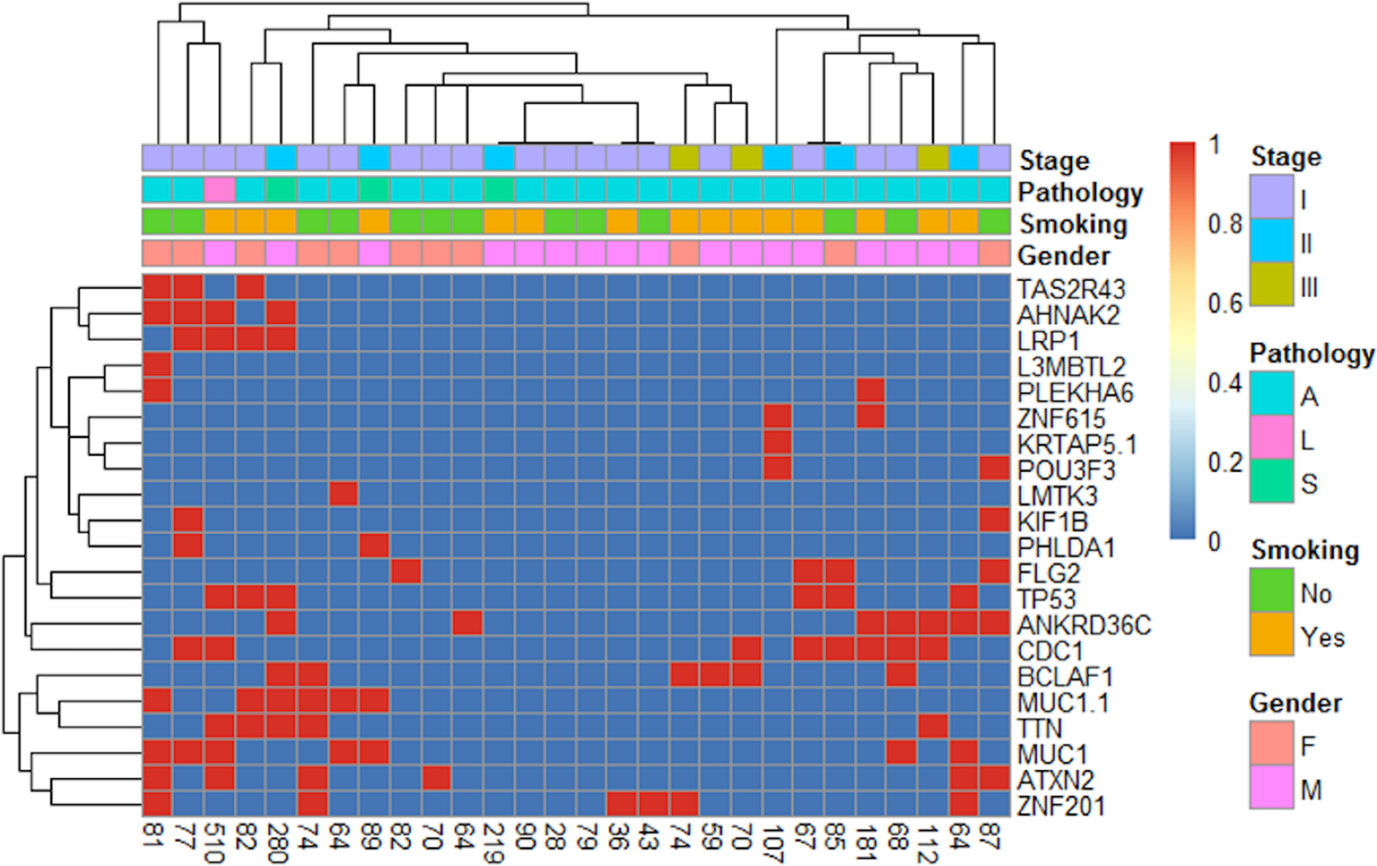
Gene heat map of different numbers of neoantigens in 28 patients.

There were 1,922 genes alterations that were associated with neoantigens, with each alternation creating 1–28 neoantigens. Among them, there were 21 genes that were associated with ≥7 neoantigens. Cluster analysis was performed for those 21 genes (**Figure 5**), and no significant correlation was observed between the number of neoantigens and the expression of those genes in each patient.

## Discussion

ICIs are more effective in patients with NSCLC with a high TMB. This has led to the suggestion that TMB might be a biomarker for predicting response to ICI treatment (16). Moreover, preclinical and clinical studies have indicated that tumor-specific missense mutations may produce particular neoantigens that mediate response to ICIs (14, 17). Accordingly, this suggests that a high TMB could lead to the production of a higher number of neoantigens and thus increase immunogenicity and result in better response to ICI treatment (18). This is further supported by our findings, which showed that non-synonymous mutations were positively correlated with the number of neoantigens. We performed additional classifications and analyses and found that the number of neoantigens was positively correlated with the presence of missense mutations (the most common mutation) and less common mutation types such as frame-shift insertions/deletions, nonsense mutations, and split-site mutations. There is evidence that frame-shift insertions or deletion are less frequent than non-synonymous single nucleotide mutations. However, they can be highly immunogenic mutations that can increase the neoantigen load and provide greater affinity for MHCs (19, 20).

Numerous studies have indicated that tumor-specific splicing is an important source of neoantigens (21–23), and although splicing frequency is relatively low, the neoantigens obtained from splicing sites are more frequent than those obtained from single-nucleotide mutations (24). We found that the frequency of nonsense mutations was lower. Nonetheless, it was also positively correlated with the number of neoantigens, indicating that nonsense mutations may produce a greater abundance of neoantigens. To the best of our knowledge, studies regarding the correlation between nonsense mutations and neoantigens have not yet been reported; therefore, further studies are needed to address this issue.

We also observed that the neoantigen load was positively correlated with base transversions and negatively correlated with base transitions. A previous study (25) of patients who received pembrolizumab revealed that patients with a durable clinical benefit were more likely to have C>A transversions and less likely to have C>T transitions (Mann-Whitney test; P=0.01). These findings are in concordance with our results.

Previous studies have used the TSNAD software to predict potential neoantigens from somatic mutations in 9,155 tumor samples from the International Cancer Genome Consortium database. They revealed that the most common potential neoantigens were encoded by KRAS, PIK3CA, and TP53. For instance, the ten most common potential neoantigens included six neoantigens derived from KRAS, which involved the G12D and G12V mutations (26). Another study of genomic, transcriptomic, and proteomic data from KRAS-mutated lung adenocarcinoma (27) identified three biological subgroups: STK11/LKB1 (KL subtype), TP53 (KP subtype), and CDKN2A/B (KC subtype). In this context, lung adenocarcinoma with the KP subtype showed a strong inflammatory response and enhanced expression of multiple co-stimulators and co-suppressors. In contrast, lung adenocarcinoma with the KL subtype expressed lower levels of immune markers. Despite similar exposures to smoking, the KP subtype had a higher mutation rate than the KL subtype, which may explain the differences in their immunogenicity (27). In our study, only three of twenty-eight patients had KRAS mutations, including one with a KRAS G12V mutation (a KP and KL mixed type) that had 85 candidate neoantigens. Another patient with a KRAS G12D mutation (the KC subtype) had 28 candidate neoantigens, and a third patient with only a KRAS G12D mutation (no combined mutations) had 87 candidate neoantigens. The number of candidate neoantigens was noticeably below the median value only in the patient with the KC subtype, while the number in the other two patients was slightly above the median value. Thus, our findings are not consistent with results reported previously regarding the correlation between KRAS mutations and neoantigens. This could be explained by the very small sample size for our analyses (only three patients with KRAS mutations).

Our study has two important limitations. First, because of financial constraints, we could not retrieve RNA-related data to guide the neoantigen prediction. Instead, this was based on the expressions of lung cancer-related genes from the TCGA database. Second, the sample size was small; therefore, a larger study will be needed to validate our findings.

In conclusion, we found that the number of candidate neoantigens was related to both the number and type of mutations. Among the mutational types, missense mutations had the highest frequency. Although less frequent, frame-shift insertions/deletions, splice site variations, and nonsense mutations were also associated with the number of candidate neoantigens, possibly because they can produce a greater abundance of neoantigens. Nevertheless, only missense mutations were positively correlated with the number of neoantigens in the multiple linear regression analysis. The number of neoantigens was positively correlated with base transversions and negatively correlated with base transitions.

## Data Availability Statement

The original contributions presented in the study are publicly available. This data can be found here: https://ngdc.cncb.ac.cn/gsa/, accession number HRA002055.

## Ethics Statement

The studies involving human participants were reviewed and approved by Ethics Committee at the Peking Union Medical College Hospital. The patients/participants provided their written informed consent to participate in this study.

## Author Contributions

HL: conceptualization, methodology, formal analysis, investigation, and writing – original draft. YX: conceptualization, investigation, writing – review and editing. MC: conceptualization, investigation, resources. JZ: conceptualization, investigation, and resources. WZ: conceptualization, investigation, and resources. XL: conceptualization, investigation, and resources. XG: conceptualization, investigation, and resources. SL: investigation and resources. JL: investigation and resources. CG: investigation and resources. HJ: software, formal analysis, and data curation. MW: conceptualization and methodology, writing – review and editing, supervision, and funding acquisition. All authors contributed to the article and approved the submitted version.

## Funding

This study was supported by the ‘13th Five-Year’ National Science and Technology Major Project for New Drugs (No: 2019ZX09734001-002), CAMS Innovation Fund for Medical Sciences (CIFMS) (to MW) (Grant No. 2018-I2M-1-003), and National Science and Technology Major Project (Grant No. 2019ZX09201-002). The funder was not involved in the study design, collection, analysis, and interpretation of data, the writing of this article or the decision to submit it for publication.

## Conflict of Interest

Author HJ was employed by company Beijing Neoantigen Biotechnology Co., Ltd.

The remaining authors declare that the research was conducted in the absence of any commercial or financial relationships that could be construed as a potential conflict of interest.

## Publisher’s Note

All claims expressed in this article are solely those of the authors and do not necessarily represent those of their affiliated organizations, or those of the publisher, the editors and the reviewers. Any product that may be evaluated in this article, or claim that may be made by its manufacturer, is not guaranteed or endorsed by the publisher.
