# Supplementary material for: Characterization of Somatic Mutations That Affect Neoantigens in Non-Small Cell Lung Cancer
Source: Front Immunol. 2022 Mar 9;12:749461. doi: 10.3389/fimmu.2021.749461 (PMC8959482; doi:10.3389/fimmu.2021.749461)
Supplement: Supplementary file 1 [file Table_1.docx]

**Table S1 Nonparametric test analysis of neoantigens**

| Variants | No. | Mean rank | Z-value | P-value |
| --- | --- | --- | --- | --- |
| Gender |  |  |  |  |
| Male | 17 | 14.97 | -0.377 | 0.720 |
| Female | 11 | 13.77 |  |  |
| Smoking History |  |  |  |  |
| No | 13 | 11.50 | -1.798 | 0.074 |
| Yes | 15 | 17.10 |  |  |
| Tumor History |  |  |  |  |
| No | 22 | 12.89 | -1.990 | 0.046 |
| Yes | 6 | 20.42 |  |  |
| Pathology |  |  |  |  |
| Squamous | 3 | 24.67 | -2.268 | 0.019 |
| Non-squamous | 25 | 13.28 |  |  |
| Clinical stage |  |  |  |  |
| I | 19 | 12.47 | - | 0.120 |
| II/III | 9 | 20.33 |  |  |
| III | 3 | 15.67 |  |  |
| Microsatellites |  |  |  |  |
| Stability | 25 | 13.74 | -1.413 | 0.173 |
| Instability | 3 | 20.83 |  |  |
| EGFR mutation |  |  |  |  |
| No | 19 | 16.34 | -1.723 | 0.087 |
| Yes | 9 | 10.61 |  |  |
